# Properties of Old Concrete Built in the Former Leipziger Palace

**DOI:** 10.3390/ma15020673

**Published:** 2022-01-17

**Authors:** Andrzej Ambroziak, Elżbieta Haustein

**Affiliations:** Faculty of Civil and Environmental Engineering, Gdansk University of Technology, 11/12 Gabriela Narutowicza Street, 80-233 Gdańsk, Poland; elzbieta.haustein@pg.edu.pl

**Keywords:** structural concrete, old concrete, building engineering, material characterization, mechanical properties, chemical properties

## Abstract

This research aims to determine the mechanical, chemical, and physical properties of old concrete used in the former Leipziger Palace in Wrocław, Poland. The cylindrical specimens were taken from the basement concrete walls using a concrete core borehole diamond drill machine. The determination of the durability and strength of old concrete was based on specified chosen properties of the old concrete obtained through the following set of tests: measurements of dry density, tests of water absorption, specification of concrete compressive strength and frost resistance, determination of the modulus of elasticity, measurement of the pH value, determination of water-soluble chloride salts and sulphate ions, and X-ray diffraction analyses. Large dispersions of the compressive strength (10.4 MPa to 34.2 MPa), density (2049 kg/m^3^ to 2205 kg/m^3^), water absorption (4.72% to 6.55%), and stabilized secant modulus of elasticity (15.25 Gpa to 19.96 GPa) were observed. The paper is intended to provide scientists, civil engineers, and designers with guidelines for examining and assessing the long-term durability of old concrete, and also extending knowledge in the field of archaeological restoration and the protection of old concrete structures.

## 1. Introduction

Concrete and reinforcement concrete were invented in the second half of the 19th century, and developed new possibilities to create new constructions and extended knowledge in civil and building engineering [1,2]. The lost knowledge of ancient concrete [3] was discovered once more and brought about new developments. The development of concrete was connected directly to the creation of new types of concrete mixes [4,5]. Proper concrete mix design and adequate placement, curation, and restriction of the influence of environmental conditions guarantee high mechanical properties [6]. Environmental conditions (sometimes unfavourable conditions) over a long time may considerably change the concrete or/and reinforcement properties. In nearly every country in the world, there are old concrete and reinforced concrete constructions that require maintenance, reconstruction, or renovation. The available tools, technology, and innovations in civil engineering should be applied in the cultural and heritage protection of old concrete and buildings structures [7]. The research and development role of the scientific and engineering community and its cooperation in the heritage protection of old structures and buildings are indispensable and key factors. Additionally, archaeological restoration and protection aspects play an important role in the protection of (not only) old concrete structures [8]. Some old constructions are demolished due to their poor technical conditions, but in most cases, due to the preservation necessity of their historical and cultural heritage, are subject to protection. To properly plan and implement preservation and protection actions, the properties of these old structures should be adequately assessed. Old concrete and reinforced concrete structures require determination of the mechanical properties in order to assess their present properties. Sometimes, additional chemical and physical properties should be determined so as to perform a proper assessment of the old structure. Many interesting scientific and engineering investigations related to the process of testing and repairing, as well as assessment of the properties of old concrete structures and constructions, have been performed. In Table 1, the chosen publications concerning investigations on old concrete structures are given. It can be seen that civil and building engineering structures made of old concrete are still being tested and investigated. It should also be noted that in active seismic regions, earthquake damage is an important causality, not only in old historical buildings [9,10], thus assessments of old concrete and reinforced concrete buildings have to be extended with earthquake knowledge [11,12,13,14]. Nevertheless, earthquake disaster damage is correlated to the types of building structures [15,16].

**Figure 1:**
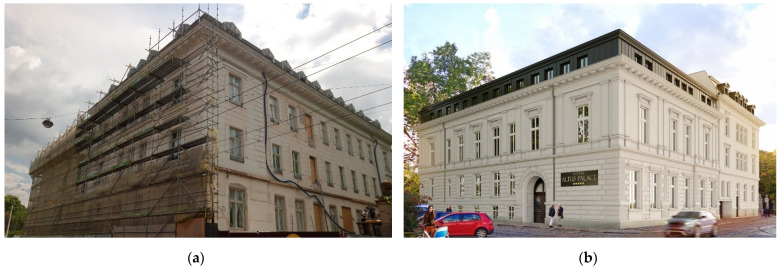
Former Leipziger Palace in Wrocław: (**a**) view on the facade during reconstruction; (**b**) facade visualization after reconstruction to the hotel building.

**Figure 2:**
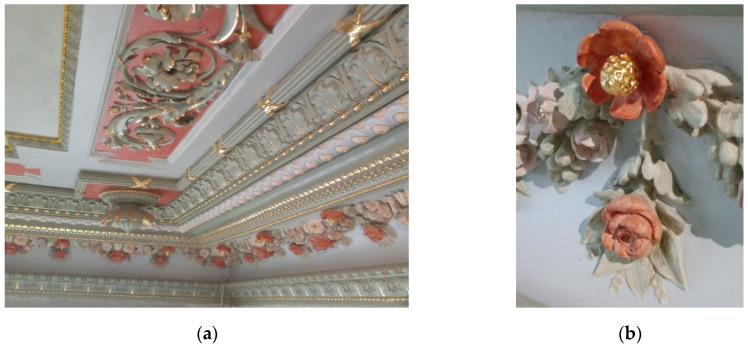
Stucco renovation on the ceiling: (**a**) partial view on the ceiling; (**b**) view of the gilded floral detail.

The present investigation of old concrete is related to a former palace building, called Leipziger Palace. The former Leipziger Palace in Wrocław (Poland) was built on the site of a riding school, from 1872 to 1874, by the banker Ignacy (Ignatz) Leipziger. The palace was designed by the famous architect Carl Schmidt (1835–1888), who was the designer of buildings important to Wrocław [52], including Hills of Partisans and the reconstruction of the Wrocław Opera. The palace was originally a two-story building with a monumental staircase located in the atrium. Representative rooms—the vestibule, living room, and dining room—were located on the first floor, and the ground floor was given a utility function. Storehouses, warehouses, a laundry room, and a porter’s apartment were arranged there. The stucco decorations were created by Theodor Milczewski’s atelier, while the cast iron decorative elements were created by Gustav Trelenberg’s company. The Leipziger had the palace for only four years, then sold the building in 1878 to the County Office (Kreisausschuss). The Palace was expanded and rebuilt in the following years. Then, the Palace served administrative functions until 1945. Next, it belonged to the Geological Company Proxima for many years. At present, the former Leipziger Palace in Wrocław is in the process of being rebuilt (by the TORUS Company from Gdańsk, Poland) into a five-star hotel, which will be named Altus Palace (see Figure 1). Currently, besides finish and building work, extensive conservation and restoration work has been carried out to restore the splendour of the old building. The architectural form of the Palace in the interior is enriched by decorations, with an antique renaissance repertoire of ornaments and decorations that have been subjected to restoration and refilling (see Figure 2).

This paper contains investigations on old concrete basement walls for which determinations of the durability and concrete strength based on the chosen mechanical, chemical, and physical properties were carried out. The concrete basement walls were 120 ÷ 140-year-old structural concrete elements. The investigation program consisted of the following sets of laboratory tests: measurements of dry density, tests of water absorption, specification of concrete compressive strength, specification of frost resistance, determination of modulus of elasticity, measurement of the pH value, determination of water-soluble chloride and sulphate ions, and carrying out X-ray diffraction analyses. It should be noted, that the available tools and laboratory equipment used to perform the present investigation were used in previous laboratory tests on old concretes performed by the authors [29,33]. The authors intend to create a database of the properties of old concrete, and thus the range of applied laboratory tests and the methods used were similar and comparable, but the conclusions and behaviours of the old concrete structures exposed to specific climatic and environmental conditions were different. The presented investigations may be treated as a part of opinions on the bearing capacity of old concrete walls and the possibilities of carrying new design loads in the extended working life for an erected five-star hotel building.

## 2. Materials and Methods

**Figure 3:**
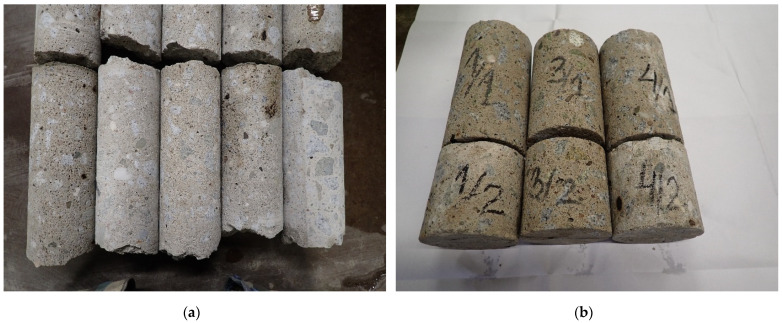
Old cylindrical concrete specimens: (**a**) taken from basement wall using a borehole diamond drill machine; (**b**) example view on some prepared concrete cores.

The old concrete specimens for the determination of the mechanical, chemical, and physical properties were taken from the structural elements of basement walls by a concrete core borehole diamond drill machine (see Figure 3a). It can be seen that some old concrete cores included visible pores (probably connected with an improper compact procedure of the concrete mix during placement) with a predominance of fine aggregate below 4mm grain-size with a low amount of additional coarse aggregates (granite and boulder) with grains with a diameter of 8 to 16 mm. Two types of concrete cores were prepared for mechanical tests (see Figure 3b). Firstly, 15 concrete cores with a diameter of approximately 100 mm (*f*_c,cycl 100_) and a length to core diameter ratio of 1 were used for compressive tests. The dimensions of the concrete cores were specified according to the standard EN 12504-1 [53]. The strength results determined for the chosen diameter of concrete cores of *f*_c,cycl 100_ can be directly comparable to the cube strength *f*_c,cube_ of 15 cm × 15 cm × 15 cm concrete specimens. Secondly, six concrete cores with a length to core diameter ratio equal to 1.5 and 2.0 with a diameter of approximately 100 mm were prepared to determine the modulus of elasticity indicated by two chosen standards. The EN 12390-13 [54] standard guidelines state that for the determination of the modulus of elasticity in compression, the ratio between the specimen length and the dimension will be in the range from 2 to 4, while the ASTM C469M standard [55] requires cores having a length-to-diameter ratio greater than 1.5. It should be noted that in old concrete structures, often it is not possible to drill cores with high length to core diameter ratios (due to, e.g., low old concrete strength, voids, or low-quality interlayers in concrete). In these cases, it is necessary to limit planned mechanical tests or to use non-standards specimen dimensions. The results obtained for cores with low dimensions can be recalculated to standard dimensions of concrete specimens according to proper guidelines with the application of the strength correction factor [56].

### 2.1. Measurements of Dry Density

The density of the old concrete was specified according to the method given in the EN 12390-7 standard [57]. Before the measure of dry density was determined, the concrete cores were stored in a ventilated oven at 105 ± 5 °C. The final concrete core mass was specified when the mass was changed by less than 0.2% in comparison with the previous mass measure. Before weighing, the old concrete specimens were cooled to room temperature in a dry, airtight vessel.

### 2.2. Tests of Water Absorption

EN 13369 standard [58] was applied to measure the water absorption of the old concrete samples. In the water absorption tests performed, the old concrete specimens were soaked in drinking water to a constant mass.

### 2.3. Determination of Concrete Compressive Strength

The Advantest 9 C300KN computer-controlled mechanical testing machine was used to perform uniaxial compressive experimental tests on the old concrete cylinder specimens. The EN 12390-3 standard [59] guidelines were applied during the concrete compressive strength tests. The mechanical testing machine with a 0.6 MPa/s constant rate of loading was used. The type and dimensions of the old concrete cylindrical specimens used were specified according to the EN 12504-1 standard [53].

### 2.4. Determination of Concrete Frost Resistance

The concrete specimens were subjected to 50 freezer cycles in a freezing chamber with a temperature- and time-controlled refrigerating and heating system according to the PN-B-06250 standard [60] for the determination of the old concrete frost resistance. The single freezer cycle consisted of freezing at −18 ± 2 °C for 4 h and thawing by total immersion in water at 18 ± 2 °C for 4 h. The old concrete can be qualified as frost resistant when the compressive strength does not decrease by more than 20% in comparison to the base samples and if, after the 50 freezer cycles, the concrete specimens are free of defects and cracks.

### 2.5. Determination of Elasticity Modulus

Determination of the secant elasticity modulus in the compression was performed using the guidelines of two standards: EN 12390-13 [54] and ASTM C469M [55]. Method B, according to EN 12390-13 [54], was applied to determine the stabilized secant modulus of elasticity *E*_C,S_ in the range of 33% ultimate strength of concrete for old core samples with a length to diameter ratio equal to 2.0. The 30% of the ultimate strength is required in ISO 1920-10 [61] for the determination of the static modulus of elasticity in compression. The diamond-drilled cores were also used in a compressometer device for measuring the static modulus of elasticity according to the ASTM C469M [55] standard guidelines. The ASTM C469M standard [55], like EN 1992-1-1 [62], needs 40% ultimate compressive strength to calculate the modulus of elasticity.

### 2.6. Determination of the pH Value

The ISO 10523 [63] standard was referred to for the determination of the old concrete pH. Before the pH value was measured, the old concrete samples were crushed and dissolved in distilled water.

### 2.7. Measurement of Water-Soluble Chloride Ions (Cl^−^) and Sulfate Ions (SO_4_^2−^)

The EN 1744-1 + A1 standard [64] was applied for the measurement of water-soluble chloride ions (Cl^−^) and sulfate ions (SO_4_^2−^). The Volhard method was used for the determination of the chloride ions. Like in the pH measurement, the old concrete samples were crushed, dissolved in distilled water, and filtrated through a mixed cellulose ester membrane filter with a pore size of 45 μm before the measurements of the water-soluble chloride ions (Cl^−^) and sulfate ions (SO_4_^2−^) were carried out.

### 2.8. Scanning Electron Microscopy (SEM) Combined with Energy Dispersive X-ray Spectroscopy (EDS)

The morphology of the old concrete samples was performed using a scanning electron microscope (SEM, type JEOL JSM 7800F, Akishima, Tokyo, Japan) equipped with an energy dispersive X-ray spectrometer (EDAX, Octane Elite, Mahwah, NJ, USA), which allowed for the identification of the elemental composition of the tested material. The accelerating voltage was 15 kV. The results of the analysis were documented with the image (SEM), the graphical representation of the reflection peaks (EDX), and the chemical composition table.

### 2.9. X-ray Diffraction (XRD) Analyses

The mineralogical composition of the concrete samples was determined by the X-ray diffractometer (XRD, MiniFlex 600, Rigaku Co., Tokyo, Japan). A copper tube (CuKα = 1.54178 Å) was used as the source of the X-ray emissions. The tests were carried out under the operating conditions of the device: 40 kV and 15 mA, 5–90 ◦2θ range, with a scanning rate of 5°·min^−1^. The study sample was grounded in an agate mortar until the whole sample passed through a 63 um sieve. PDXL software was used to process the diffraction data.

## 3. Laboratory Test Results and Discussion

### 3.1. Measurement of Dry Density

**Figure 4:**
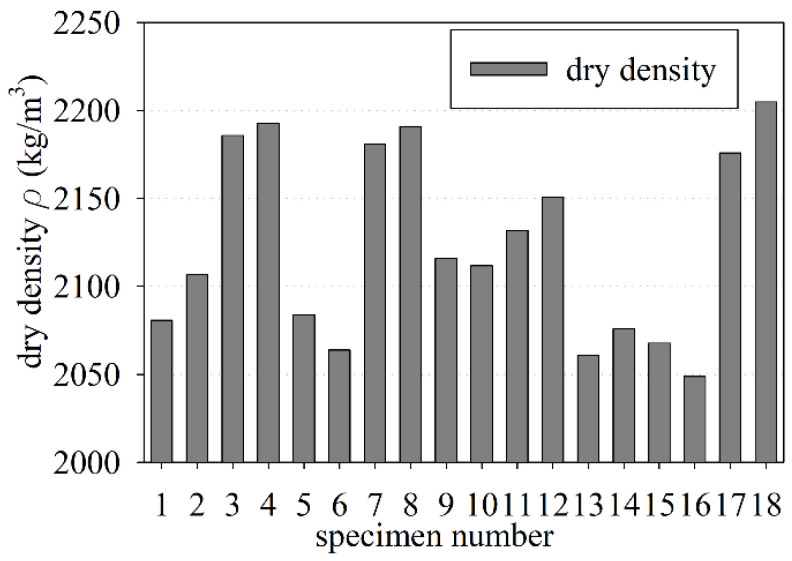
Measurement of the old concrete dry density.

The laboratory tests showed that the dry density of the old concrete varied from 2049 kg/m^3^ to 2205 kg/m^3^ (see Figure 4). The mean dry density value of the old concrete was equal to 2124 ± 13 kg/m^3^, while the median was 2114 kg/m^3^. The mean dry density value fulfilled the conditions for normal-weight concrete, according to EN 206 [65] (the dry density over 2000 kg/m^3^ and below to 2600 kg/m^3^). However, the mean dry density of the old concrete specimens are below the 2160 kg/m^3^ limit specified in the ACI 318-19 standard [66] for normal-weight concrete; thus, the investigated old concrete could not be categorized as normal concrete, according to the ACI 318-19 standard [66].

### 3.2. Measurement of Water Uptake Capacity (Water Absorption Test)

**Figure 5:**
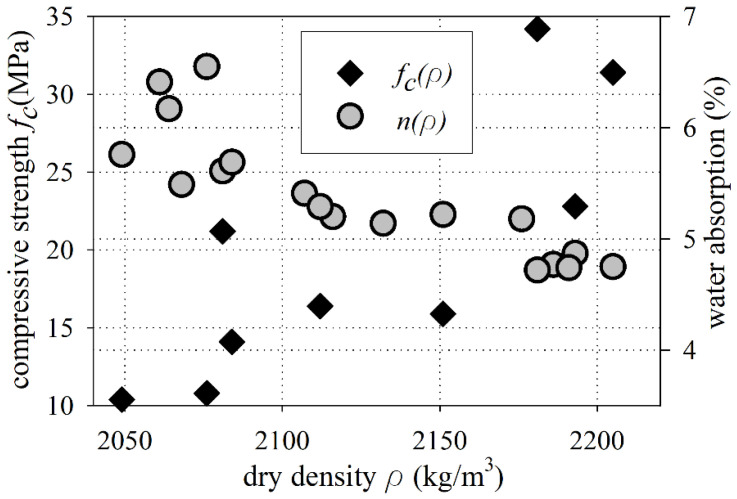
Compressive strength and water absorption vs. dry density.

The specified water uptake capacity of the old concrete samples ranged from 4.72 to 6.55% (see Figure 5). The mean value of the water absorption was 5.39 ± 0.13%, while the median was equal to 5.26%. The water absorption for structural concrete should not be greater than 9% for concrete protected from atmospheric conditions, and not greater than 5% for concrete exposed to atmospheric conditions [60]. On the other hand, the structural concrete is qualified as poor quality when the water absorption value is greater than 5%, according to the report of the International Federation for Structural Concrete [67]. The high water absorption of concrete causes problems through the decrease in concrete durability [68].

### 3.3. Old Concrete Compressive Strength

**Figure 6:**
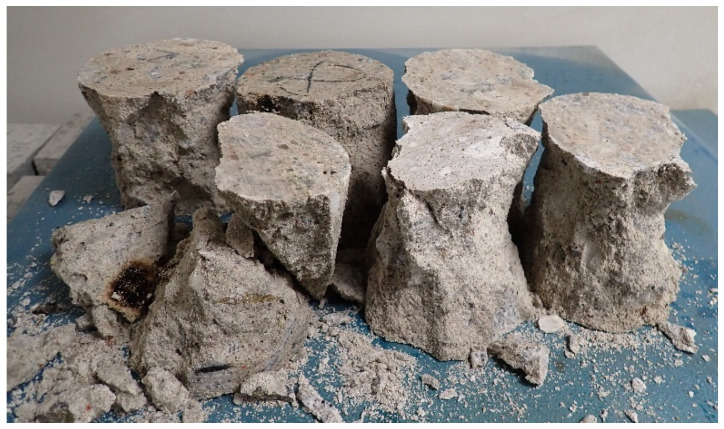
Parts of some concrete specimens after uniaxial compressive tests—views of the form of failure with visible organic inclusions and predominance of fine aggregates.

The uniaxial test results of the compressive strength for old concrete cores vs. the dry density are shown in Figure 5. Figure 5 exhibits the general characteristic, that an increase in the density of old concrete caused a higher compressive strength and lower water absorption of old concrete. The compressive strength of concrete cores varied from 10.4 MPa to 34.2 MPa. The difference in compressive strength results may result from the production technology, which was probably based on hand mixing with handmade proportions of the concrete components. Additionally, a lack of uniform compaction during the placement of old concrete causes differences in dry density and compressive strengths. The mean in-situ compressive strength value was 19.7 ± 2.8 MPa, while the median was equal to 16.4 MPa. The strength results of the concrete cores were comparable to the cube strength (see EN 12504-1 standard [53]), so *f*_c,cube_ = *f*_c,cycl 100_ = 19.7 ± 2.8 MPa. Figure 6 shows that parts of some concrete specimens after uniaxial compressive tests—views of the form of failure with visible organic inclusions and predominance of fine aggregates.

Then, we compared the determined mean in-situ compressive strength values with the regulations, guidelines, and investigations performed on old concretes from the period of erection and rebuild for the former Leipziger Palace. Paulík [17] reported, for the Monier type arch concrete bridge completed in 1892, an average concrete cube compressive strength from 25.8 MPa to 29.9 MPa, with an average bulk density of 2080 to 2140 kg/m^3^. Hallauer [69] indicated a compressive strength equal to 18 ± 7 MPa for concrete building in a structural element of a river canal built in 1896–1900, and 6.63 ± 2.45 MPa for the concrete elements of a pier built in 1908–1919. Hallauer [69] also emphasised that the forecast compressive strength was about 15–18 MPa after 28 days and 18–24.5 MPa after 45 days for Hennebique recommended concrete mixtures. Hellebois and Espion [26] stated that concrete built in 1904 in Colo-Hugues viaduct (Belgium) possessed compressive strength from 19.7 MPa to 54.2 MPa. Wolert et al. [23], for the bridge over Barnes Slough and Jenkins Creek (USA), built between 1914 and 1916, determined the compressive strength to be from 12.1 to 23.0 MPa. The German Committee for Structural Concrete [70], in 1916, indicated two concrete strength classes of 14.7 MPa and 17.7 MPa for usage in concrete structures. The Regulations on the Construction and Maintenance of Road Bridges [71], from January 1926, forecasted the cube compressive strength of concrete from about 5.9 MPa to 19.6 MPa with an amount of cement from 100 kg to 500 kg, respectively, related to 1 m^3^ aggregate in the concrete mixes. The PN-B-195 standard [72], issued in 1934, defined the extremal characteristic strength of concrete as being equal to 16.7 MPa, with 400 kg cement at a 1:2 ratio of sand to gravel in 1 m^3^ parts of mixed concrete. The present strength of the old concrete built in basement walls with a mean compressive strength of 19.7 ± 2.8 MPa, was similar to concrete structures built during this time. Nevertheless, it should be noted that old concrete strength variations are related directly to cement and the aggregate types used in the mixes, placement and compact technology, curation, and environmental conditions during and after the placement process of the concrete mixes [6,73].

Finally, it is possible to determine the characteristic in-situ compressive cube strength *f*_ck,is,cube_ of concrete in an old structure according to the EN 13791 [74] standard, which is given by the following:

(1) $$f_{\mathrm{ck},\mathrm{is},\mathrm{cube}}=\min\left\{\begin{array}{c} f_{m,\mathrm{is}}-k_{n}\cdot s\\ f_{\mathrm{is},\mathrm{lowest}}+M \end{array}\right\}=\min\left\{\begin{array}{c} 19.7-1.96\times 2.8\\ 10.4+1 \end{array}\right\}=\min\left\{\begin{array}{c} 14.2\\ 11.4 \end{array}\right\}=11.4\;\mathrm{MPa},$$

where *f*_m,is_ is the mean I -situ cube compressive strength, $f_{\mathrm{is},\mathrm{lowest}}$ is the lowest in situ cube compressive strength test results, *k*_n_ is the factor that depends on the number of test results, *s* is the standard deviation of the in situ compressive strength, and *M* is the value of margin. The C8/10 compressive strength class according to EN 206 [65] is estimated for the old concrete structure, based on the specified characteristics of the in situ cube compressive strength of *f*_ck,is,cube_ = 11.4 MPa. The C8/10 compressive strength class, according to the EN 206 standard [65], should have a minimum characteristic cylinder strength (*f*_ck,cyl_) of 8 MPa (N/mm^2^) and cube strength (*f*_ck,cube_) of 10 MPa.

### 3.4. Old Concrete Frost Resistance

**Figure 7:**
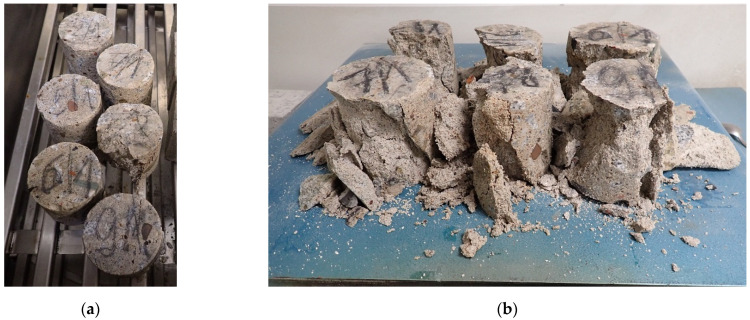
Frost resistance tests: (**a**) view of concrete specimens inside the freezing chamber after tests; (**b**) view of a form of failure concrete specimens.

**Figure 8:**
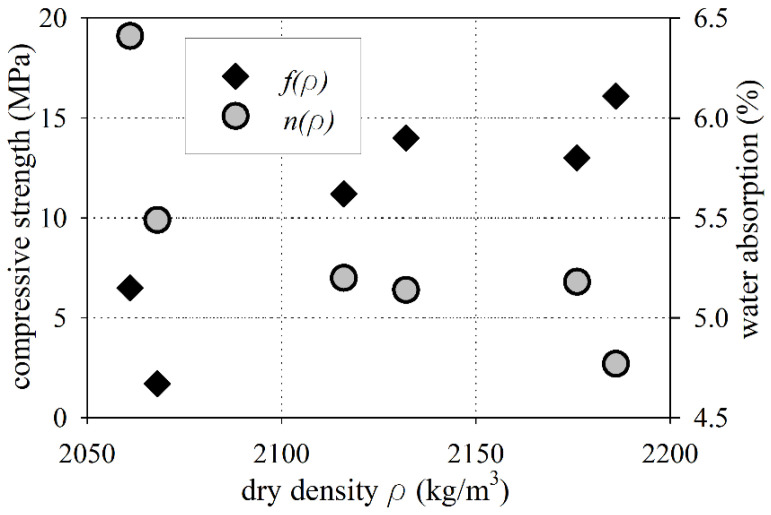
Compressive strength after 50 freezer cycles and water absorption vs. dry density.

Cylindrical samples were subjected to 50 freezer cycles consisting of freezing at −18 ± 2 °C for 4 h and thawing by total immersion in water at 18 ± 2 °C for 4 h. After 50 freezer cycles (see Figure 7a), the compressive strength tests were performed (see Figure 7b). The compressive strength of the old concrete after 50 freezer cycles varied from 1.7 MPa to 16.1 MPa. The mean value of the compressive strength after 50 freezer cycles was 10.4 ± 2.2 MPa, while the median was 12.1 MPa (see Figure 8). The value of the mean compressive strength after 50 freezer cycles was about 47% lower than the compressive strength of the base samples ($f_{m,\mathrm{is}}$ = 19.7 MPa). Additionally, two cores showed cracks and visible loosening parts in the concrete from samples (see Figure 7a). The difference between compressive strength, before and after 50 freezer cycles, was considerably higher than 20%, and cracks appeared on the old concrete cores. Therefore, according to PN-B-06250 [60], the old concrete did not have freezing resistance properties.

### 3.5. Modulus of Elasticity in Compression

**Figure 9:**
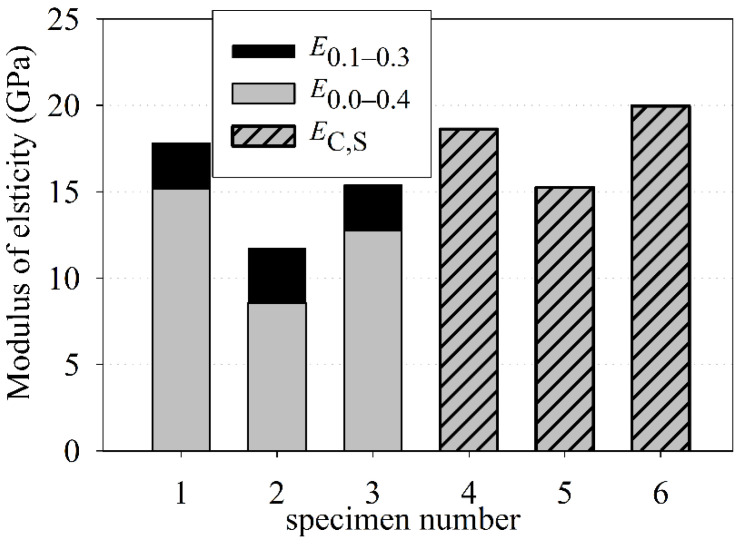
Modulus of elasticity in compression vs. dry density.

The stabilized secant modulus of elasticity, *E*_C,S_, specified according to EN 12390-13 [54], ranged from 15.25 to 19.96 GPa (see Figure 9), with a mean value equal to 17.95 GPa. For concrete cores tested according to ASTM C469M [55], the moduli of elasticity was specified as the applicable customary working stress, ranging from 0 to 40% and from 10 to 30%. A difference of about 18% between the mean values of the modulus of elasticity for *E*_0.0–0.4_ and *E*_0.1–0.3_ was observed. The mean modulus of elasticity for *E*_0.0–0.4_ was 12.17 GPa and for *E*_0.1–0.3_ was 14.97 GPa.

The present EN 1992-2 standard [62] states that for the C8/10 concrete strength class, the secant modulus of elasticity can be assumed as 25 GPa. The guidelines [71], issued near 100 years earlier, indicate that for concrete compressive strengths lower than 9.81 MPa (100 kg/cm^2^) and greater or equal to 13.73 MPa (140 kg/cm^2^), the modulus of elasticity corresponds values of 9810 MPa (100,000 kg/cm^2^) and 14,715 MPa (150,000 kg/cm^2^), respectively. For the specified characteristic in-situ cube compressive strength equal to 11.4 MPa, the modulus of elasticity corresponded to a value 11.8 GPa, according to guidelines [71]. Nevertheless, the modulus of elasticity mainly depends on the properties of the aggregates used in the concrete mixes and on the concrete strength class. The aggregate with a predominance of fine aggregate with a high sand point was used in the investigated old concrete mix.

### 3.6. Determination of the pH Value

**Figure 10:**
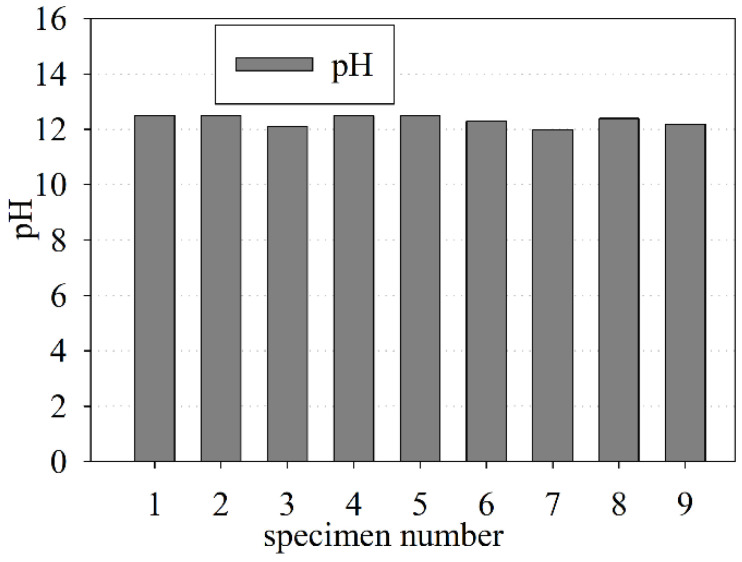
The pH value of the old concrete.

The pH value is an important factor that influences the corrosion rate of reinforcement [75,76]. Freshly made concrete has a pH value that varies from 12.5 to 13.5 [77], while the corrosion of reinforcement generally occurs when the pH value is less than 9 [78]. Under the laboratory tests for the old concrete samples, it was determined that the mean value of the pH was 12.33 ± 0.06, while the median was 12.40. The pH values for the old concrete ranged from 12.00 to 12.50 (see Figure 10). The pH of the old concrete was in the safety range. One of the concrete cores (which was not subjected to strength tests) included a flat bar-shaped reinforcement, which was in a very good condition, which confirms the results obtained for the safe pH value.

### 3.7. Measurement of Water-Soluble Chloride Ions (Cl^−^) and Sulfate Ions (SO_4_^2−^)

**Figure 11:**
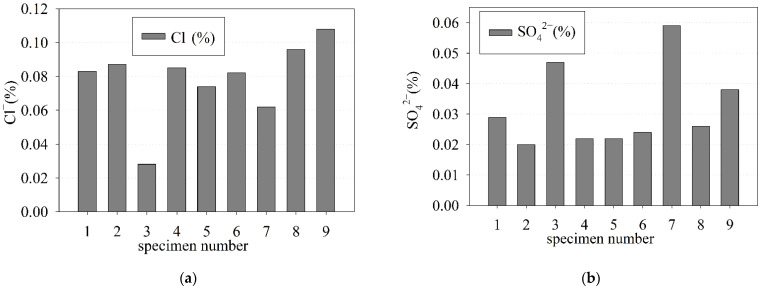
Content as the dry weight percentage of water-soluble (**a**) chloride ions (Cl^−^); (**b**) sulfate ions (SO_4_^2−^).

The content of water-soluble chloride ions (Cl^−^) and sulfate ions (SO_4_^2−^) was determined as the percentage of dry weight using the chemical tests. The water-soluble chloride ion values ranged from 0.028 % to 0.108 % (see Figure 11a), with the mean value equal to 0.078 ± 0.008% and the median equal to 0.083 %. The sulfate ion values ranged from 0.020 to 0.059% dry weight (see Figure 11b), while the mean value was 0.032 ± 0.004% and the median was 0.026% dry weight.

The present standards and guidelines give chloride ions and sulfate ions limits as a percentage of the mass of cement, therefore, it is necessary to convert the percentage content of chloride ions and sulfate ions from the dry weight percentage to the percentage mass of cement. The mean percentage content of chloride ions by mass of cement was about 0.5%, and considerably exceeded the limits for reinforced concrete stated by the EN 206 standard [65] (0.2%) and the ACI 318 code [79] (0.15%), respectively. The increased content of chloride ions in the tested specimens of concrete was probably strongly affected by the C_3_A increased content in the cement used in the preparation of the old concrete mix [80], and the influx or rising of water containing salt compounds into old concrete. The mean percentage content of sulfate ions by mass of cement was 0.2%, and did not exceed 4% (limit guidelines by BS 8110-1:1985 standard [81]). Based on the obtained results, it can be concluded that the old concrete was not exposed to sulfate attack.

### 3.8. Results of Scanning Electron Microscopy (SEM) Combined with Energy Dispersive X-ray Spectroscopy (EDS)

**Figure 12:**
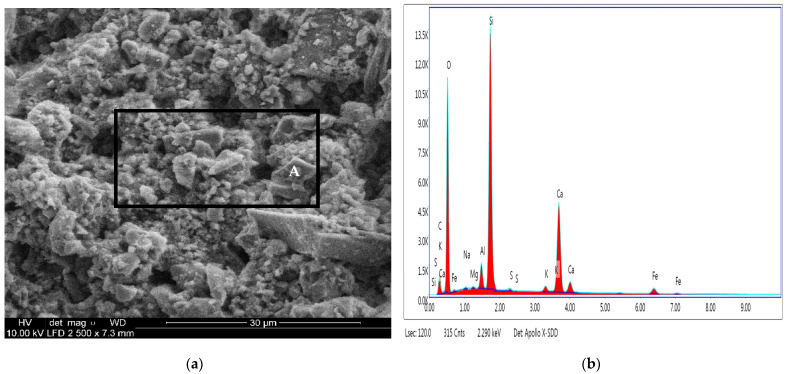
SEM/EXD analysis: (**a**) SEM microscopic images; (**b**) EDS of the sample on the selected area (A).

The scanning electron microscopy (SEM) image of the chosen sample taken from the old concrete structure is shown in Figure 12a. The results of the analysis of the chemical composition of the sample (in the selected area marked in Figure 12a) based on the presented spectrum (see Figure 12b) determined by the EDAX method are collected in Table 2.

The presented image SEM (see Figure 12a) of the chosen test sample shows that the old concrete element was a layer structure formed from the products of the cement hydration process. The shape and size of the crystalline phases are the result of the formation of chemical compounds in the structure of the tested concrete. According to the data presented in Table 2, the content of elements in the tested sample, taken from the structural old concrete element, is as follows: silica (43.9%), calcium (41.2%), iron (8.3%), aluminium (3.9%), and potassium (1.9%) by weight, respectively. The remaining amount of the identified elements in the structure of the tested sample was below 1% of the mass of the concrete sample.

### 3.9. X-ray Diffraction Analyses

**Figure 13:**
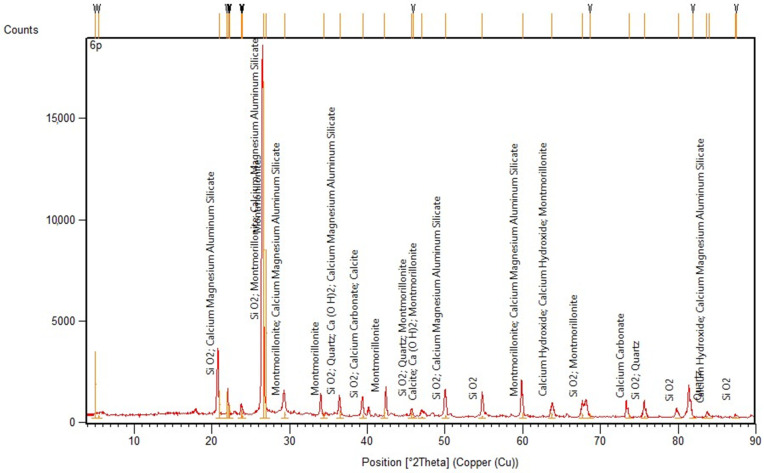
XRD pattern of the old concrete sample.

The XRD method was applied for the identification of the phase composition of the old concrete. The results of the mineralogical composition (in the form of a diffraction pattern) based on a chosen old concrete sample are presented in Figure 13.

The analysis of the evolution of concrete over the years, its phase composition, and its microstructure is critical to determine its durability. The obtained test results (see Figure 13) indicate that the following compounds were mainly dominant in the concrete structure after 120 to 140 years: (Na,Ca)_0,3_(Al,Mg)_2_ (Si_4_O_10_) (OH)_2_xnH_2_O–montmorillonite, calcium–magnesium–aluminium–silicate hydrate (C-M-A-S-H), CaCO_3_ (calcite) as the carbonate phase, Ca(OH)_2_–portlandite, and SiO_2_–quartz. The registered chemical compounds are products of the reacted clinker phases included in the cement used for concrete, including many years of exposure to atmospheric conditions and water.

The calcium–silicate–hydrate (C-S-H) is the most important of the hydration phases of cement. C-S-H precipitates from the ions produced in the pore solution through the dissolution of anhydrous calcium silicates (C_3_S or C_2_S), but also from other soluble siliceous materials that may be present in the cement. In the investigated old concrete samples, the calcium silicate was combined with aluminium and magnesium ions. The present form is the result of long-term environmental influences. The crystalline CaCO_3_ is the result of the reaction of atmospheric carbon dioxide with the mineral components of the cement. With CO_2_ entering the concrete for many years, carbonation reactions can produce one or more reaction fronts that progressively move through the concrete structure. The resulting carbonation products influence the porosity/permeability of concrete. The inward diffusion of carbonate ions forms calcite, which stabilizes the paste and develops a carbonate-stabilized shell. The presence of a chemical that forms complex compounds that include calcium, magnesium, and aluminium ions may result from the hydration process of the cement used for concrete, or it may be the result of the influence of a corrosive environment on the structural concrete for 120 to 140 years.

## 4. Conclusions

This paper focuses mainly on the characterization of old concrete samples from a historical building. The mechanical, chemical, and physical properties of old concrete built in the former Leipziger Palace in Wrocław, Poland, were specified through laboratory tests. The investigation found the following conclusions:

- The mean dry density value equalled 2114 kg/m^3^ and fulfilled the requirements for normal weight concrete.
- The mean value of the water absorption of old concrete equalled 5.39 ± 0.13%, and this concrete could be categorized as having a poor concrete quality.
- The determined characteristic in situ compressive cube strength *f*_ck,is,cube_ of the concrete in the old structure equalled 11.4 MPa. while the mean in situ compressive strength value was 19.7 ± 2.8 MPa.
- The C8/10 compressive strength class was estimated for the old concrete basement walls structures.
- The old concrete did not have freezing resistance properties due to a more than 20% difference between the compressive strength before and after 50 freezer cycles, and because cracks appeared on the old concrete cores after 50 freezer cycles.
- The mean value of the stabilized secant modulus of elasticity *E*_C,S_ was 17.95 GPa.
- The mean value of the pH of the investigated old concrete was 12.33 ± 0.06 and was in the safety range.
- The changes in the chemical and mineral composition of the concrete structure had an impact on its porosity/permeability as a function of the time of exposure to atmospheric conditions and water.
- Taking into account the data, the content of chloride and sulphate ions were 0.03 ± 0.11 and 0.02 ± 0.06% dry weight in the concrete, respectively. The obtained values were the result of the chemical processes occurring in the concrete.
- The chemical composition (SEM/EDS) and X-ray composition (XRD) indicated the domination of certain compounds in the old concrete structure, such as calcium-magnesium–aluminium–silicate hydrate (C-M-A-S-H), CaCO_3_ (calcite), and Ca(OH)_2_–portlandite.
- The old concrete had a layer structure formed from the products of the cement hydration process.
- The registered chemical compounds were the products of the reacted clinker phases included in the cement used for concrete, including many years of exposure to the atmospheric conditions and water.
- The old concrete did not meet most of the current standard requirements (see Appendix A, Table A1), nevertheless, after proper protection and strengthening of the old building structural system, it could carry new design loads for an extended working life.

This paper determined the properties of old concrete built in the former Leipziger Palace nearly 120 to 140 years ago. Determination of the mechanical, chemical, and physical properties of old concrete makes it possible for proper future investigations on planning for the condition assessment of old concrete constructions. The authors are hopeful that the present investigation sparks interest among a wide group of engineers, archaeologists, and scientists on the subject of old concrete. This investigation may also be treated as motivation for new investigations on old concrete structures.

## Figures and Tables

**Table 1:** References of investigations into the chosen old concrete structures.

Investigation Subject	References	Location
123-year-old concrete bridge	Paulík [17]	Slovakia
107-year-old bridge	Rabiega et al. [18]	Poland
104-year-old bridge	Sena-Cruz et al. [19]	Portugal
100-year-old bridge	Słomka-Słupik et al. [20]	Poland
100-year-old reinforced concrete dome	Onysyk et al. [21]	Poland
100-year-old bridge	Witzany and Zigler [22]	Czech Republic
100-year-old reinforced concrete flat slab bridge	Wolert et al. [23]	USA
100-year-old reinforced concrete viaduct	Jóźwiak-Niedźwiecka and Tucholsk [24]	Poland
95-year-old viaduct	Hellebois et al. ([25,26])	Belgium
95-year-old concrete dam	Blanco et al. [27]	Spain
95-year-old concrete arch bridge	Ambroziak and Malinowski [28], Ambroziak et al. [29]	Poland
90-year old concrete mortar	Trägårdh and Lagerblad [30]	Sweden
84-year-old reinforced concrete bridge	Gebauer and Harni [31]	Switzerland
80-year-old reinforced concrete structure	Melchers and Chaves [32]	Australia
70-years-old concrete office building	Ambroziak et al. [33]	Poland
63-year-old reinforced concrete promenade	Melchers et al. [34]	Scotland
60-year-old concrete pier	Castro-Borges et al. [35]	Mexico
60-years-old reinforced concrete elevated water tanks	Dilena et al. [36]	Italy
57-years-old concrete viaducts	Medeiros-Junior et al. [37]	Brazil
50-years-old reinforced concrete trough bridge	Richard et al. [38]	Sweden
48-year-old concrete bridge girders	Pettigrew et al. [39]	USA
45-year-old Sorell Causeway bridge	Papé and Melchers [40]	Australia
40-year-old reinforced concrete beams	Dasar et al. [41]	Japan
40-year-old concrete bridge girder	Czaderski and Motavalli [42]	Switzerland
30-to-50-year-old concrete structures	Sohail et al. [43]	Arabian Gulf region
20-year-old concrete office building	Qazweeni and Daoud [44]	Kuwait
28-year-old reinforced concrete arch ribs	Zhang et al. [45]	China
28-year-old concrete	Prassianakis and Giokas [46]	Greece
26-year-old reinforced concrete beam	Khan et al. [47]	France
10-year-old concrete	Chen [48]	China
10-year-old crumb rubber concrete bridge deck	Zhu et al. [49]	China
5-year-old concrete prepared with recycled aggregates	Kou and Poon [50]	China
4-year-old mortar cement	Dasar et al. [51]	Japan

**Table 2:** Element compositions of the sample determined via X-ray EDS.

Type of Element	Mg	Al	Si	S	K	Na	Ca	Fe
%	0.24	3.94	43.85	0.39	1.93	0.22	41.18	8.25

## Data Availability

All of the laboratory test result data are included in Tables and Figures in the present paper. Upon request, the numerical version of the results will be provided.

## Appendix A

The comparison of the chosen properties for the old investigated concrete built in basement walls and the requirements for new concrete are collected in Table A1.

**Table A1:** Comparison between the chosen properties for the investigated old concrete and requirements for the new concrete.

Properties	Investigated Old Concrete	Requirements for New Concrete
concrete strength class	C8/10	min. C16/20 or C30/37 for XC2 or XA1 exposure class
dry density	2124 kg/m^3^	about 2300 kg/m^3^
water absorption	5.26%	below 4%
frost resistance	not passed	yes
modulus of elasticity	17.95 GPa	29–32 GPa
pH value	12.33	12.5 to 13.5
chloride ions by mass of cement	0.5%	below 0.2%

## References

[1] Moussard M., Garibaldi P., Curbach M. (2017). The invention of Reinforced concrete (1848—1906). Proceedings of the High Tech Concrete: Where Technology and Engineering Meet—Proceedings of the 2017 fib Symposium.

[2] Jahren P., Sui T. (2018). History of Concrete a Very Old and Modern Material.

[3] Bao Y., Zhang Y.-S. (2003). RESEARCH OF 4500-YEAR-OLD CONCRETE. Role of Concrete in Sustainable Development.

[4] Lydon F.D. (1982). Concrete Mix Design.

[5] Ziolkowski P., Niedostatkiewicz M. (2019). Machine Learning Techniques in Concrete Mix Design. Materials.

[6] Ambroziak A., Ziolkowski P. (2020). Concrete compressive strength under changing environmental conditions during placement processes. Materials.

[7] Cruz A., Coffey V., Chan T.H.T., Perovic M. (2021). Engineering in heritage conservation. J. Cult. Herit. Manag. Sustain. Dev..

[8] Faber K.T., Casadio F., Masic A., Robbiola L., Walton M. (2021). Looking Back, Looking Forward: Materials Science in Art, Archaeology, and Art Conservation. Annu. Rev. Mater. Res..

[9] Hadzima-Nyarko M., Mišetić V., Morić D. (2017). Seismic vulnerability assessment of an old historical masonry building in Osijek, Croatia, using Damage Index. J. Cult. Herit..

[10] Liel A.B., Lynch K.P. (2012). Vulnerability of Reinforced-Concrete-Frame Buildings and Their Occupants in the 2009 L’Aquila, Italy, Earthquake. Nat. Hazards Rev..

[11] Mase L.Z., Likitlersuang S., Tobita T. (2021). Ground Motion Parameters and Resonance Effect During Strong Earthquake in Northern Thailand. Geotech. Geol. Eng..

[12] Stręk A.M., Lasowicz N., Kwiecień A., Zajac B., Jankowski R. (2021). Highly dissipative materials for damage protection against earthquake-induced structural pounding. Materials.

[13] Khatami S.M., Naderpour H., Mortezaei A., Razavi S.M.N., Lasowicz N., Jankowski R. (2021). Effective gap size index for determination of optimum separation distance preventing pounding between buildings during earthquakes. Appl. Sci..

[14] Tanapalungkorn W., Mase L.Z., Latcharote P., Likitlersuang S. (2020). Verification of attenuation models based on strong ground motion data in Northern Thailand. Soil Dyn. Earthq. Eng..

[15] Ahmed M.S., Morita H. (2018). An analysis of housing structures’ earthquake vulnerability in two parts of Dhaka city. Sustain..

[16] Tsang H.-H., Wenzel F. (2016). Setting structural safety requirement for controlling earthquake mortality risk. Saf. Sci..

[17] Paulík P., Bačuvčík M., Brodňan M., Koteš P., Vičan J. (2016). Reconstruction of the Oldest Reinforced Concrete Bridge in Slovakia in Krásno nad Kysucou. Procedia Eng..

[18] Rabiega J., Sadowski K., Biliszczuk J.H.P. (2005). Evaluation of bearing capacity of Zwierzyniecki Bridge across old Odra river in Wrocław (Ocena nośności mostu Zwierzynieckiego nad Starą Odrą we Wrocławiu). Inżynieria I Bud..

[19] Sena-Cruz J., Ferreira R.M., Ramos L.F., Fernandes F., Miranda T., Castro F. (2013). Luiz bandeira bridge: Assessment of a historical reinforced concrete (RC) bridge. Int. J. Archit. Herit..

[20] Słomka-Słupik B., Podwórny J., Grynkiewicz-Bylina B., Salamak M., Bartoszek B., Drzyzga W., Maksara M. (2021). Concrete examination of 100-year-old bridge structure above the kłodnica river flowing through the agglomeration of upper silesia in gliwice: A case study. Materials.

[21] Onysyk J., Biliszczuk J., Prabucki P., Sadowski K., Toczkiewicz R. (2014). Strengthening the 100-year-old reinforced concrete dome of the Centennial Hall in Wrocław. Struct. Concr..

[22] Witzany J., Zigler R. (2020). Rehabilitation Design of a Historic Concrete Arch Bridge in Prague from the Early 20th Century. J. Perform. Constr. Facil..

[23] Wolert P.J., Kolodziejczyk M.K., Stallings J.M., Nowak A.S. (2020). Non-destructive Testing of a 100-Year-Old Reinforced Concrete Flat Slab Bridge. Front. Built Environ..

[24] Jóźwiak-Niedźwiecka D., Tucholski Z. (2010). Reinforced concrete viaduct from begining of the 20th century—Microstructure analysis of 100 years old concrete (Wiadukt żelbetowy z początków XX wieku—Analiza mikrostruktury stuletniego betonu). Drog. I Most..

[25] Hellebois A., Launoy A., Pierre C., De Lanève M., Espion B. (2013). 100-year-old Hennebique concrete, from composition to performance. Constr. Build. Mater..

[26] Hellebois A., Espion B. (2011). Concrete properties of a 1904 Hennebique reinforced concrete viaduct. WIT Trans. Built Environ..

[27] Blanco A., Segura I., Cavalaro S.H.P., Chinchón-Payá S., Aguado A. (2016). Sand-Cement Concrete in the Century-Old Camarasa Dam. J. Perform. Constr. Facil..

[28] Ambroziak A., Malinowski M. (2021). A 95-Year-Old Concrete Arch Bridge: From Materials Characterization to Structural Analysis. Materials.

[29] Ambroziak A., Haustein E., Niedostatkiewicz M. (2020). Chemical, Physical, and Mechanical Properties of 95-Year-Old Concrete Built-In Arch Bridge. Materials.

[30] Trägårdh J., Lagerblad B. (1998). Leaching of 90-Year Old Concrete Mortar in Contact with Stagnant Water.

[31] Gebauer J., Harnik A.B. (1975). Microstructure and composition of the hydrated cement paste of an 84 year old concrete bridge construction. Cem. Concr. Res..

[32] Melchers R.E., Chaves I.A. (2021). Durable Steel-Reinforced Concrete Structures for Marine Environments. Sustainability.

[33] Ambroziak A., Haustein E., Kondrat J. (2019). Chemical and Mechanical Properties of 70-Year-Old Concrete. J. Mater. Civ. Eng..

[34] Melchers R.E., Li C.Q., Davison M.A. (2009). Observations and analysis of a 63-year-old reinforced concrete promenade railing exposed to the North Sea. Mag. Concr. Res..

[35] Castro-Borges P., De Rincón O.T., Moreno E.I., Torres-Acosta A.A., Martínez-Madrid M., Knudsen A. (2002). Performance of a 60-year-old concrete pier with stainless steel reinforcement. Mater. Perform..

[36] Dilena M., Dell’Oste M.F., Gubana A., Morassi A., Polentarutti F., Puntel E. (2021). Structural survey of old reinforced concrete elevated water tanks in an earthquake-prone area. Eng. Struct..

[37] Medeiros-Junior R.A., Lima M.G., Yazigi R., Medeiros M.H.F. (2015). Carbonation depth in 57 years old concrete structures. Steel Compos. Struct..

[38] Richard B., Epaillard S., Cremona C., Elfgren L., Adelaide L. (2010). Nonlinear finite element analysis of a 50 years old reinforced concrete trough bridge. Eng. Struct..

[39] Pettigrew C.S., Barr P.J., Maguire M., Halling M.W. (2016). Behavior of 48-Year-Old Double-Tee Bridge Girders Made with Lightweight Concrete. J. Bridg. Eng..

[40] Papé T.M., Melchers R.E. (2011). The effects of corrosion on 45-year-old pre-stressed concrete bridge beams. Struct. Infrastruct. Eng..

[41] Dasar A., Hamada H., Sagawa Y., Yamamoto D. (2017). Deterioration progress and performance reduction of 40-year-old reinforced concrete beams in natural corrosion environments. Constr. Build. Mater..

[42] Czaderski C., Motavalli M. (2007). 40-Year-old full-scale concrete bridge girder strengthened with prestressed CFRP plates anchored using gradient method. Compos. Part B Eng..

[43] Sohail M.G., Kahraman R., Ozerkan N.G., Alnuaimi N.A., Gencturk B., Dawood M., Belarbi A. (2018). Reinforced Concrete Degradation in the Harsh Climates of the Arabian Gulf: Field Study on 30-to-50-Year-Old Structures. J. Perform. Constr. Facil..

[44] Qazweeni J., Daoud O. (1991). Concrete deterioration in a 20-years-old structure in Kuwait. Cem. Concr. Res..

[45] Zhang J., Li C., Xu F., Yu X. (2007). Test and Analysis for Ultimate Load-Carrying Capacity of Existing Reinforced Concrete Arch Ribs. J. Bridg. Eng..

[46] Prassianakis I.N., Giokas P. (2003). Mechanical properties of old concrete using destructive and ultrasonic non-destructive testing methods. Mag. Concr. Res..

[47] Khan I., François R., Castel A. (2012). Structural performance of a 26-year-old corroded reinforced concrete beam. Eur. J. Environ. Civ. Eng..

[48] Chen C., Chen X., Li X. (2020). Dynamic Compressive Behavior of 10-Year-Old Concrete Cores after Exposure to High Temperatures. J. Mater. Civ. Eng..

[49] Zhu H., Duan F., Shao J., Shi W., Lin Z. (2019). Material and durability study of a 10-year old crumb rubber concrete bridge deck in Tianjin China. Mag. Concr. Res..

[50] Kou S.-C., Poon C.-S. (2008). Mechanical properties of 5-year-old concrete prepared with recycled aggregates obtained from three different sources. Mag. Concr. Res..

[51] Dasar A., Patah D., Hamada H., Sagawa Y., Yamamoto D. (2020). Applicability of seawater as a mixing and curing agent in 4-year-old concrete. Constr. Build. Mater..

[52] Grzegorczyk B., Tomaszkiewicz A. (2000). Wrocławskie pałace czynszowe Carla Schmidta i Friedricha Barchewitza powstałe w latach siedemdziesiątych XIX stulecia. Rocz. Wrocławski.

[53] CEN (European Committee for Standardization) (2009). EN 12504-1 Testing Concrete in Structures—Part 1: Cored Specimens—Taking, Examining and Testing in Compression.

[54] CEN (European Committee for Standardization) (2013). EN 12390-13 Testing Hardened Concrete—Part 13: Determination of Secant Modulus of Elasticity in Compression.

[55] (2014). ASTM C469M-14 Standard Test Method for Static Modulus of Elasticity and Poisson’s Ratio of Concrete in Compression.

[56] Khoury S., Aliabdo A.A.H., Ghazy A. (2014). Reliability of core test—Critical assessment and proposed new approach. Alexandria Eng. J..

[57] CEN (European Committee for Standardization) (2019). EN 12390-7 Testing Hardened Concrete—Part 7: Density of Hardened Concrete.

[58] CEN (European Committee for Standardization) (2001). EN 13369 Common Rules for Precast Concrete Products.

[59] CEN (European Committee for Standardization) (2019). EN 12390-3 Testing Hardened Concrete. Compressive Strength of Test Specimens.

[60] (1988). PN-88/B-06250 Normal Concrete.

[61] ISO (International Organization for Standardization) (2010). ISO 1920-10 Testing of Concrete—Part 10: Determination of Static Modulus of Elasticity in Compression.

[62] CEN (European Committee for Standardization) (2004). EN 1992-1-1 Eurocode 2: Design of Concrete Structures—Part 1-1: General Rules and Rules for Buildings..

[63] (2008). ISO 10523 Water Quality—Determination of pH.

[64] CEN (European Committee for Standardization) (2009). EN 1744-1:2009+A1 Tests for Chemical Properties of Aggregates—Part 1: Chemical Analysis.

[65] CEN (European Committee for Standardization) (2016). EN 206:2013+A1:2016 Concrete—Specification, Performance, Production and Conformity.

[66] ACI (American Concrete Institute) (2019). Building Code Requirements for Structural Concrete.

[67] (1989). Report No. 192. Diagnosis and Assessment of Concrete Structures—State-of-Art.

[68] Zhang S.P., Zong L. (2014). Evaluation of relationship between water absorption and durability of concrete materials. Adv. Mater. Sci. Eng..

[69] Hallauer O. (1989). Die Entwicklung der Zusammensetzung von Beton für Wasserbauten. Mtteilungsblaetter Bundesanst. Wasserbaut..

[70] (1916). Bestimmungen für die Ausführung von Bauwerken aus Eisenbeton.

[71] (1926). Rules for the Construction and Maintenance of Road Bridges (Przepisy o Budowie i Utrzymaniu Mostów Drogowych).

[72] (1934). Structural Analysis and Design.

[73] Petrounias P., Giannakopoulou P., Rogkala A., Stamatis P., Lampropoulou P., Tsikouras B., Hatzipanagiotou K. (2018). The Effect of Petrographic Characteristics and Physico-Mechanical Properties of Aggregates on the Quality of Concrete. Minerals.

[74] CEN (European Committee for Standardization) (2019). EN 13791 Assessment of In-Situ Compressive Strength in Structures and Precast Concrete Components.

[75] Stojanović G., Radovanović M., Krstić D., Ignjatović I., Dragaš J., Carević V. (2019). Determination of pH in powdered concrete samples or in suspension. Appl. Sci..

[76] Abdulrahman A.S., Mohammad I., Mohammad S.H. (2011). Corrosion inhibitors for steel reinforcement in concrete: A review. Sci. Res. Essays.

[77] Duffó G.S., Farina S.B., Giordano C.M. (2009). Characterization of solid embeddable reference electrodes for corrosion monitoring in reinforced concrete structures. Electrochim. Acta.

[78] Hansson C.M., Poursaee A., Jaffer S.J. (2012). Corrosion of Reinforcing Steel Bars in Concrete. Masterbuilder.

[79] ACI (American Concrete Institute) (2014). ACI 318-14 Building Code Requirements for Structural Concrete.

[80] Kim M.J., Kim K.B., Ann K.Y. (2016). The influence of C3A content in cement on the chloride transport. Adv. Mater. Sci. Eng..

[81] (1985). BS 8110-1:1985 Structural Use of Concrete. Code of Practice for Design and Construction.

